# Locating the Sex Determining Region of Linkage Group 12 of Guppy (*Poecilia reticulata*)

**DOI:** 10.1534/g3.120.401573

**Published:** 2020-08-04

**Authors:** Deborah Charlesworth, Roberta Bergero, Chay Graham, Jim Gardner, Lengxob Yong

**Affiliations:** * Institute of Evolutionary Biology, School of Biological Sciences, University of Edinburgh, West Mains Road, EH9 3LF, UK; † University of Cambridge, Department of Biochemistry, Sanger Building, 80 Tennis Ct Rd, Cambridge CB2 1GA, UK; ‡ Centre for Ecology and Conservation, University of Exeter, Penryn, Falmouth, Cornwall, TR10 9FE, UK

**Keywords:** Duplication, linkage disequilibrium, genome assembly, partial sex linkage

## Abstract

Despite over 100 years of study, the location of the fully sex-linked region of the guppy (*Poecilia reticulata*) carrying the male-determining locus, and the regions where the XY pair recombine, remain unclear. Previous population genomics studies to determine these regions used small samples from recently bottlenecked captive populations, which increase the false positive rate of associations between individuals’ sexes and SNPs. Using new data from multiple natural populations, we show that a recently proposed candidate for this species’ male-determining gene is probably not completely sex-linked, leaving the maleness factor still unidentified. Variants in the chromosome 12 region carrying the candidate gene sometimes show linkage disequilibrium with the sex-determining factor, but no consistently male-specific variant has yet been found. Our genetic mapping with molecular markers spread across chromosome 12 confirms that this is the guppy XY pair. We describe two families with recombinants between the X and Y chromosomes, which confirm that the male-determining locus is in the region identified by all previous studies, near the terminal pseudo-autosomal region (PAR), which crosses over at a very high rate in males. We correct the PAR marker order, and assign two unplaced scaffolds to the PAR. We also detect a duplication, with one copy in the male-determining region, explaining signals of sex linkage in a more proximal region.

The sex chromosome pair of the guppy have been studied for many decades with the aim of understanding the evolution of recombination suppression between Y and X chromosomes. The earliest genetic studies in the guppy discovered sex linkage of male coloration traits that are polymorphic in guppy natural populations (Winge 1922b; Winge 1922a), and numerous such traits in natural and captive populations have been shown to be transmitted from fathers to sons in this fish, with only a few major coloration factors being autosomal (Haskins and Haskins 1951; Haskins *et al.* 1961; Lindholm and Breden 2002). Coloration factors show complete or (more rarely) partial sex-linkage disproportionately often, given that this fish has 23 chromosome pairs (Nanda *et al.* 2014), and the sex chromosome pair represents about 3.8% of the genome (Künstner *et al.* 2017). This observation strongly suggests that these factors are sexually antagonistic (SA), based on the following reasoning (Bull 1983). SA mutations can occur in autosomal and sex-linked genes, creating conflicts between the sexes. If the conflict is resolved by evolving sex-specific expression, any selection favoring sex linkage is abolished. The observation that guppy male-coloration genes are frequently sex-linked therefore suggests that selection formerly either favored the evolution of sex linkage, or, more likely, that sex linkage favors establishment of polymorphisms for SA mutations. SA effects of male coloration factors are supported by evidence from studies in natural populations of Trinidadian guppies (including Haskins *et al.* 1961; Gordon *et al.* 2012). It has therefore been thought likely that SA polymorphisms may have been involved in selecting for suppressed recombination between the two members of this chromosome pair (reviewed in Wright *et al.* 2017; Bergero *et al.* 2019).

Cytogenetic and genetic studies have consistently demonstrated strong genetic sex-determination in all Trinidadian guppy (*P. reticulata*) samples studied, from various source rivers and domesticated strains (Tripathi *et al.* 2009; Nanda *et al.* 2014; Künstner *et al.* 2017; Wright *et al.* 2017; Bergero *et al.* 2019; Dor *et al.* 2019; Winge 1922a; Winge 1922b) including natural population males (Haskins *et al.* 1961). Chromosome 12 was later identified as the XY pair (Tripathi *et al.* 2009; Nanda *et al.* 2014; Künstner *et al.* 2017; Wright *et al.* 2017; Bergero *et al.* 2019; Dor *et al.* 2019), and the same sex chromosome is found in the close relative, the Venezuelan gupp*y*, *P. wingei* (Nanda *et al.* 2014; Darolti *et al.* 2020). XX male or XY female individuals are very rare in guppies (Winge and Ditlevsen 1947). The situation therefore differs from that in the zebrafish, where a genetic sex determinant is detected in wild samples, but not domesticated strains (Howe *et al.* 2013; Bellott *et al.* 2014).

Figure 1 shows a schematic diagram indicating, in general terms, the regions where crossovers are observed on the guppy XY pair. This is an acrocentric chromosome pair, and shows little heteromorphism (Winge 1922b), though differences between the Y and X have been detected between *P. wingei* strains, and the one captive Trinidadian guppy sample studied differed from *P. wingei* (Nanda *et al.* 2014). A sex-linked region occupying considerably less than half of the sex chromosome pair has been identified, based on male-specific heterochromatin and staining by male-specific probes in FISH experiments, and the Y sometimes appears longer than the X (Nanda *et al.* 1990; Traut and Winking 2001; Lisachov *et al.* 2015). Using MLH1 focus detection on chromosomes in sperm cells Lisachov *et al.* (2015) found localization of crossovers at the termini most distant from the centromeres of the guppy XY pair and the autosomes. The chromosomes are acrocentric, and the mean number of MLH1 foci in a testis cell was 23.2 ± 0.5, representing a single crossover on each chromosome on average (Lisachov *et al.* 2015). Genetic mapping with molecular markers also suggested that crossovers on the sex chromosome pair in male meiosis occur mainly in the terminal region, and rarely nearer the centromere (Tripathi *et al.* 2009); the crossover events were not localized physically, but 12 out of 13 events in males occurred in the terminal one-sixth of the chromosome, whereas all 15 events in meiosis of the females were in the 4 centromere-proximal sixths (a significant difference by Fisher’s Exact test, *P* = 0.0001). Lisachov *et al.* (2015) also observed crossover events in male meiosis in a terminal part of the XY pair just centromere-proximal to the male-specific LG12 region mentioned above, which probably includes the sex-determining locus (Figure 1; the size of this region is not yet certain, as explained below). It is also not known whether the changes between regions with different recombination rates are gradual or represent sharp transitions, like those at some mammalian PAR boundaries (Marais and Galtier 2003; Van Laere *et al.* 2008).

**Figure 1:**
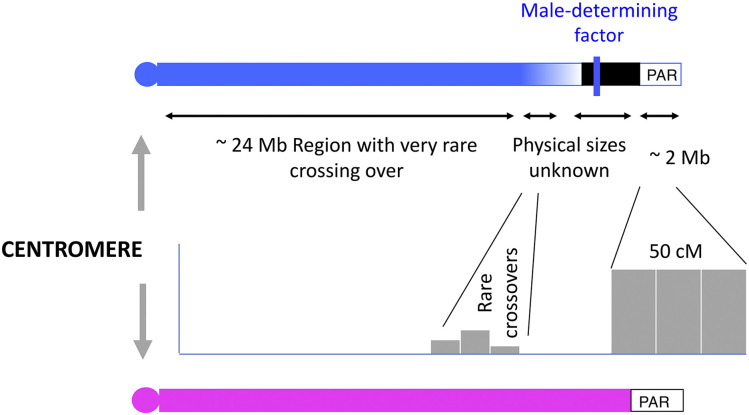
Schematic diagram of the *P. reticulata* XY sex chromosome pair (with the X in pink) to show regions in which crossovers in males probably occur at different rates (symbolized by the bar charts), based on cytogenetic data and population genomic results (see Introduction). Much or all of the chromosome is present on both the X and Y, and coverage of sequences is very similar in the two sexes [the Y is not degenerated and appears not to have lost genes carried by the X (see (Bergero *et al.* 2019)]. The diagram shows the centromere at the left-hand end (as in the genome assembly), and the PAR, where most crossover events occur, at the right-hand end. The sizes of the regions indicated are not yet accurately known, and the diagram shows the current information. The region at the centromeric end that has extremely low crossing over in male meiosis may extend across much of the XY pair, and the highly recombining PAR appears to be at most 2 Mb in size (Bergero *et al.* 2019). The Results section below describes evidence that the male-determining locus (symbolized by a blue vertical line) is just proximal to the PAR boundary at around 25.7 Mb. The region carrying the male-determining locus may lie within a completely non-recombining male-specific Y region (or MSY), as the presence of male-specific heterochromatin suggests (see Introduction); if so, the region could also include other genes, so the diagram shows a physically extended MSY (in black; this region may be missing from the female assembly, and therefore remain undetected, since such male-specific sequences would not have mapped to the female reference genome). It remains possible that there is just a very small maleness factor, for example a single gene or SNP, as discussed in the Introduction section.

However, although population genomic studies confirm that chromosome 12 is the XY pair, they do not identify an extensive fully sex-linked region (Bergero *et al.* 2019; Darolti *et al.* 2020). Instead, variants across much of the chromosome show weak associations with the sex-determining region (Wright *et al.* 2017; Bergero *et al.* 2019). There is evidence for migration of sequence variants between the X and Y chromosomes in the large centromere-proximal region, strongly suggesting that rare recombination occurs in this region, as well as in the terminal pseudo-autosomal region, labeled PAR in Figure 1, where most crossovers occur (Bergero *et al.* 2019; Darolti *et al.* 2020). If recombination occurs in male meiosis in addition to the high rate of crossovers in the terminal PAR, this will prevent fixation of male-specific variants in the recombining regions, and can account for the low differentiation and low divergence between the sexes in the guppy, across most of chromosome 12 observed in both those studies, and also by (Darolti *et al.* 2020) and in the present study (see below). Figure 1 therefore indicates the possibility of rare crossover events in the region proximal to the sex-determining region.

Specifically, the figure indicates a region where (Nanda *et al.* 1990; Lisachov *et al.* 2015) detected such events cytologically, although their analysis could not precisely determine its size or location. Clearly, the occurrence of even rare crossovers, in even a limited region proximal to the sex-determining region, allow sequence variants to move between the X and Y chromosomes; differentiation and divergence will thus establish an equilibrium low level, rather than increasing to ever higher levels over evolutionary time. The guppy XY pair therefore differs from XY pairs of many well-studied organisms. In humans, birds and some plants, divergence at synonymous sites (reflecting the number of generations of isolation of the Y from the X) is much higher than the value of about 1–2% estimated in the guppy (Darolti *et al.* 2020), and clearly distinct divergence levels, called “evolutionary strata”, are detectable (Lahn and Page 1999; Skaletsky *et al.* 2003; Zhou *et al.* 2014). The oldest stratum must carry the sex-determining locus, and, as expected, is found in different mammals (Cortez *et al.* 2014), birds (Wang *et al.* 2014) and snakes (Schield *et al.* 2019; Schield *et al.* 2020) that share the same sex-determining locus, while the younger strata are inferred to have evolved later. The guppy may have a “stratum” that includes its sex-determining locus, and this probably corresponds to the male-specific region described above. However, the evidence just described that its XY pair occasionally recombine makes it unlikely that any younger strata have evolved. Even rare X-Y recombination will prevent the establishment of diverged Y-linked strata carrying Y-specific variants; even if crossovers occur only in a restricted region, any diverged Y sequences will recombine in female meiosis after they have crossed over into a female.

Further family studies are, however, needed to refine our understanding of the guppy sex-determining region, and to determine whether this is an extensive, multi-gene fully sex-linked region or stratum, or a small region, potentially a single gene like the insertion of the maleness factor in the medaka, *Oryzias latipes* (Kondo *et al.* 2009), or even a single SNP, as has been inferred in another fish, the tiger pufferfish or Fugu, *Takifugu rubripes* (Kamiya *et al.* 2012). Our study advances understanding of the guppy sex-determining region’s location, and of the regions that recombine between the XY pair.

It was recently reported that a candidate for the sex-determining region has been mapped on linkage group 12 of the guppy (Dor *et al.* 2019). Genotypes of a microsatellite marker named *gu1066* within the Stomatin-like 2 (*STOML2*) gene, were found to be associated with the sexes of fish from two domesticated guppy strains, Red Blonde and Flame. This marker is located at 25,311,467 bp in the published assembly of the guppy LG12 (Künstner *et al.* 2017), in the region where the cytogenetic results just outlined suggest the presence of the sex-determining locus. These authors also identified a candidate for the guppy sex-determining gene within a 0.9 Mbp region near this marker. Of 27 annotated genes in this region, the *GADD45G-like* gene (at 24,968,682 bp in the LG12 assembly) was identified as a good candidate for the guppy sex-determining gene, or at least for having with a role in male fertility, although the sequence did not include any male-specific alleles and its expression was not sex-biased (Dor *et al.* 2019).

Here, we show that the *gu1066* microsatellite does not have male-specific alleles in natural populations of Trinidadian guppies, and that a polymorphic SNP (single nucleotide polymorphism) in the candidate gene has no male-specific allele. We also describe new mapping results using multiple microsatellite and SNP genetic markers that that revealed Y-X recombinants that provide the first direct genetic evidence that the location of the sex-determining locus is within the region suggested by the previous studies cited above, including Dor *et al.* (2019).

## Methods

### Fish samples, DNA extraction

Supplementary Table S1 describes the 14 natural populations samples analyzed here, and the sources of the fish used for genetic mapping. These were sampled from natural populations in Trinidad in February 2017, and most individuals were photographed as live specimens in the field before being killed and preserved. Live fish from the same natural populations were also transported to the United Kingdom, and maintained at the University of Exeter, Falmouth, for genetic studies (see below).

Genomic DNA for microsatellite genotyping, genotyping of intron length variants, and for high-throughput genotyping (see below), was extracted using the Echolution Tissue DNA Kit (BioEcho, Germany). Microsatellite markers ascertained from the guppy complete genome sequence assembly were genotyped as described previously (Bergero *et al.* 2019). The primers are listed in Supplementary Table S2, along with the primers for the *gu1066* microsatellite.

### Genetic mapping and high-throughput genotyping

Because guppy females breed best when kept together with other individuals, some of the “families” whose genotypes are reported here were generated from multiple parental individuals, and the parents of five of the six full sibships described below were distinguished using microsatellite markers. The QHPG5 family was generated from two females housed with one male, and the PMLPB2 family from three females and two males; only the LAH family involved a single male and single female.

Using sequences from the guppy female whole genome sequence assembly (Künstner *et al.* 2017), we identified genic sequences found in all 16 Trinidadian guppy individuals sampled from a natural population (10 males and 6 females), and ascertained SNPs across LG12 from our own resequencing of these individuals (Bergero *et al.* 2019). These SNPs were targetted for high-throughput genotyping (SeqSNP) experiments with genomic DNA extracted as described above. The experiments were carried out by LGC Genomics (LGC Genomics GmbH, Ostendstraße 25, 12459 Berlin, Germany, www.lgcgroup.com/genomics). The targetted SNPs were selected from within coding sequences, with the criterion that about 50 bp of sequence flanking each such SNP should also be coding sequence, in order to maximize the chance that the sequence would amplify in diverse populations, and to minimize the representation of SNPs in repetitive sequences. To further avoid repetitive sequences, the SNPs were chosen to avoid ones whose frequencies in the ascertainment sample were 0.5 in both sexes. The SNPs and their locations in the guppy genome assembly are listed in a Supplementary Table S10, together with some features of the mapping results. As expected, the primers worked well for most targeted sequences.

Genetic mapping was done as described previously, using microsatellites ascertained from the guppy complete genome sequence assembly, as well as these SNPs. The set of SNPs included one within the candidate gene, *GADD45G-like*, identified by Dor *et al.* (2019); the gene is also named LOC103474023, and starts at position 24,968,682 in the guppy LG12 assembly (Künstner *et al.* 2017).

### Estimation of F_ST_ values between the sexes

F_ST_ values of individual SNPs between males and females were estimated separately for each population. The sites with variants differed between the samples from the different natural population samples listed in Supplementary Table S1. For each site that varied in a population, the F_ST_ value was computed following (Hudson *et al.* 1992), to quantify the proportion of the total diversity in a sample of sequences that is found between the samples of males and females from each of the natural populations. The *F_ST_* value expected for a fully sex-linked SNP (with all males heterozygous, and all females homozygous for one of these variants) depends on the number of individuals of each sex that were sampled (Bergero *et al.* 2019; Darolti *et al.* 2020); with the samples listed in Supplementary Table S1, this value is about 1/3^rd^.

### Analyses to search for potentially sex-linked genes in guppy unplaced scaffolds

In order to try and include the whole of the guppy sex chromosome, we also identified two unplaced scaffolds, NW_007615023.1 and NW_007615031.1, that are located near one end of the assembly of the *Xiphophorus maculatus* (platyfish) chromosome, Xm8, which is homologous to the guppy LG12 (Amores *et al.* 2014). The platyfish is a close relative of the guppy (Pollux *et al.* 2014), with a mean synonymous site divergence of around 10%; for comparison, this is similar to the value for *Drosophila melanogaster* and *D. simulans* (Tamura *et al.* 2004).

To search for such orthologs, we did reciprocal-best hit BLAT searches using as query the coding sequences (cds) of genes assembled on chromosome 8 of the platyfish (the homolog of the guppy sex chromosome pair) as an initial query against all cds found in the guppy. This identified two unplaced scaffolds that include multiple genes (the complete results are shown in the file “Unlocalised_Contigs_UNLOCALISED_Scaffolds_LG12” deposited in Dryad). NW_007615031.1 has a total length of ∼325 kb, and is ∼5-8 kb from the start of the Xm8 assembly (which corresponds with the non-centromere end of the guppy LG12 assembly). This is annotated with 32 genes. NW_007615023.1 is a ∼490 kb scaffold that is assembled 1.4 -1.6 Mb from the Xm8 start, and is annotated with 22 genes and a pseudogene (23 genes in Ensembl). We identified and mapped microsatellites within these scaffolds.

### Data availability

Supplementary material available at figshare: https://doi.org/10.25387/g3.12739289.

## Results

### No complete association with maleness for markers in the GADD45G-Like gene region

We genotyped the *gu1066* microsatellite in three natural high predation populations of Trinidadian guppies. 16 individuals (32 alleles) of each sex were sampled from each population, and a total of 29 different alleles were observed, of which 11 were shared between two or more population samples (Supplementary Tables S3 and S4). None of the alleles showed any association with the sexes of the individuals, and no alleles, other than occasional rare alleles (mostly singletons), were male-specific in any of the populations sampled. No results consistent with complete Y-linkage were seen. For example, allele 264 was seen only in 4 males from the Guanapo population, and one male from the Yarra population, but it is homozygous in one of the Guanapo males, showing that it can be carried on the X as well as the Y chromosome. Our results from these natural populations show that this region is not strongly associated with the sex-determining locus of the guppy, and suggest that the observed association is most likely due to use of a captive sample.

A polymorphic SNP in the candidate gene identified by Dor *et al.* (2019) is, however, associated with maleness, although no allele is fully male-specific. We genotyped this SNP in 14 natural populations, and it was polymorphic in six of them. Table 1 shows the results from these six populations pooled (the full details, including the genotypes, are in Supplementary Table S5). A Fisher’s exact test shows that, overall, males are heterozygous significantly more often than females (*P* = 0.0004). However, only 17% of the males genotyped are heterozygotes (Table 1). Possible interpretations of these findings are in the Discussion section below.

**Table 1:** Genotypes at an SNP at position 24,969,110 bp in the female assembly, within the candidate sex-determining gene, *GADD45G-like* (or LOC103474023) in two high-throughput genotyping experiments (the experiment number is indicated in column 1). The river from which each population sample was derived is given, and HP and LP denote high- and low-predation sites, respectively. Part A shows the results for each of the 14 populations sampled (see Supplementary Table S5 for more details), and part B summarizes the overall association of the variant with the male phenotype

A: Genotypes observed in the samples					
Experiment number	Population	Numbers of individuals			
		Males	Females	Males heterozygous for the variant	Homozygous females (variant not present, %)
1	Guanapo LP (GLZ)	14	10	3	100
1	Guanapo HP	14	10	No variation	
1	Marianne HP	17	7	No variation	
1	Marianne LP	14	10	No variation	
1	Yarra HP	14	10	No variation	
1	Yarra LP	14	10	No variation	
2	Aripo HP, AH	14	10	1	100
2	Aripo LP, AL	14	10	3	100
2	Quare LP	16	10	No variation	
2	Quare HP	16	10	1	100
2	Turure LP	16	10	6	10
2	Turure HP	16	10	1	100
2	Paria LP	8	16	No variation	
2	Guanapo LP (GLT)	5	5	No variation	
**B: Totals across the six populations with variants present**					
				**Heterozygous**	**Homozygous**
		**Total in males**		15	75
		**Total in females**		0	60

### Analysis of F_ST_ values between the sexes in the same natural population samples

**Figure 2:**
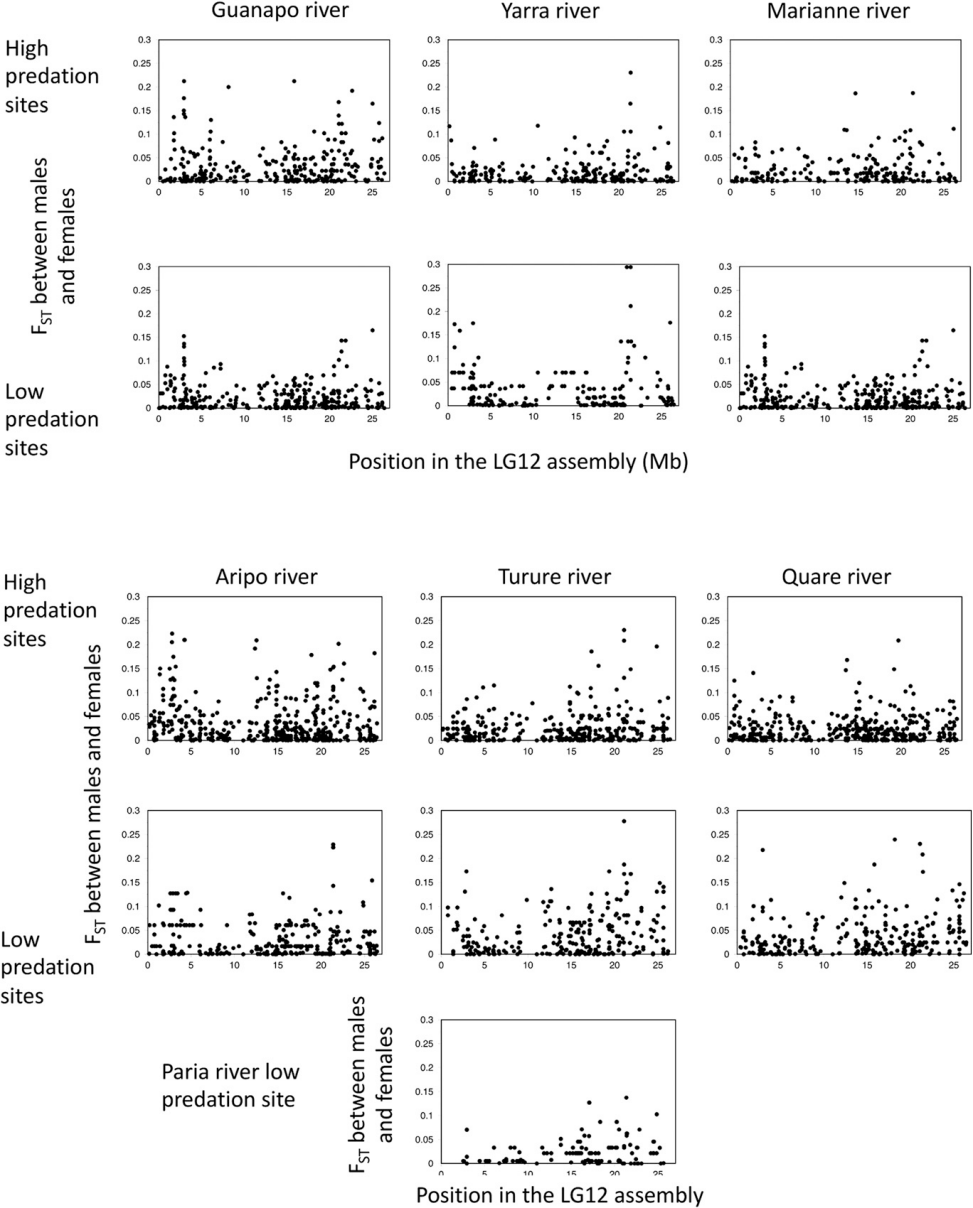
Differentiation between males and females sampled from natural populations from rivers in the Northern range of Trinidad (Supplementary Table S1), based on high-throughput genotyping results for LG12 SNPs. Differentiation is estimated as the F_ST_ value within each population, which is expected to be about 0.3 for fully sex-linked sites, and the y axes of the plot for each population extend up to this value; no SNPs with higher F_ST_ values were observed in our samples. Sites with high and low predation rates were sampled from each river, except for the Paria river, where there are no high predation sites.

14 of the natural populations listed in Supplementary Table S1 were genotyped for LG12 SNPs to test for fully sex-linked genotype configurations. Figure 2 shows that, although associations are detected by our analysis, they are not complete. In these natural populations, no region consistently has numerous SNPs showing differentiation between the sexes near the F_ST_ value of around 0.3 expected for fully sex-linked variants; furthermore, both heterozygotes and homozygotes are found in both sexes. This includes

the apparently fully sex-linked SNPs previously described in a captive population (Bergero *et al.* 2019). Similarly to our previous results, F_ST_ values between the sexes are often highest in the region proximal to 5 Mb in the published female assembly of the chromosome, and, to a lesser extent, across the region distal to 20 Mb, where the sex-determining locus is thought to be located (see Figure 1). Similar associations with the sexes scattered across LG12 were found in genome sequences of samples of 10 fish of each sex from natural populations from the Yarra, Quare and Aripo rivers (Almeida *et al.* 2020). However, as discussed below, genetic mapping and other results show that the signals of associations with the sex-determining locus seen in the centromere-proximal region are not evidence that the sex-determining locus is located in a proximal part of LG12.

### Genetic mapping in families to detect the PAR boundary and X-Y recombination events

Both the male and female genetic maps are similar in the different populations, which include all three drainages in Trinidad. Supplementary Figure S1 shows genetic mapping results for the families reported previously (Bergero *et al.* 2019), some with more markers, plus some other families newly mapped here, whose source populations are listed in Supplementary Table S1. As for the families studied previously, almost all crossovers in male meiosis were within the physically small terminal LG12 region pseudo-autosomal region.

Out of 721 individuals of known sex that we have genotyped for genetic mapping in eleven *P. reticulata* families (Supplementary Table S1), only two recombinants were detected in any LG12 region other than the PAR. One male in family QHPG5 was a recombinant, and one female in family PMLPB2. The dams and sires of both families were sampled alive from natural populations in Trinidad and the progeny were generated in the United Kingdom (see Methods).

The PMLPB2 family (from the Petit Marianne river, in the Northern drainage of the Trinidad Northern mountain range, see Willing *et al.* 2010) varied at seven LG12 microsatellite markers (Supplementary Table S6); out of 69 female and 68 male progeny genotyped, the recombinant female (PMLPB2_f23r) carries the sire’s Y-linked alleles at the 4 more proximal LG12 markers, located from 1.2 to 11.7 Mb in the published assembly, but his X-linked alleles at the 3 distal markers, including a marker at 21.3 Mb. The sex-determining locus cannot therefore be proximal to 11.7 Mb Mb. Unfortunately, no variable marker was found in the sire of this family between 11.7 and 21.3 Mb.

**Figure 3:**
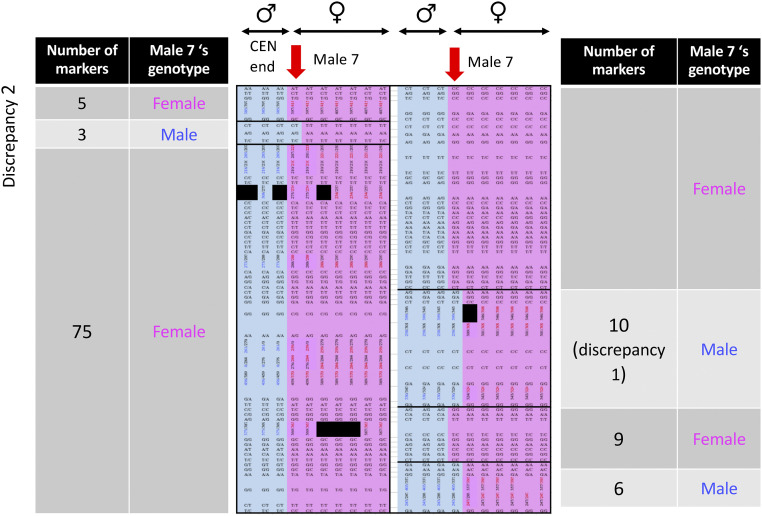
Summary of genotypes at non-PAR markers informative in male meiosis in sibship 2 from the Quare QHPG5 family, in diagrammatic form, showing the two discrepancies between these male genetic map results and the female genome assembly (see also Table 1 and full details, including the marker names and locations, in Supplementary Table S7). Each column shows the genotypes of a single progeny individual in the sibship, with males at the left and females at the right, and the colors indicate whether, for a given marker, an individual received the sire’s X- or Y-linked allele marker (genotypes with paternal X- linked alleles are colored in pink, and genotypes with paternal Y-linked alleles are in blue). The left-hand part shows the 58 more centromere-proximal markers, and the right-hand part shows the 58 more terminal markers, up to the inferred PAR boundary. 108 markers that co-segregate perfectly with the individuals’ sexes in meiosis of the male parent of the 13 sibship 1 progeny, with the same sire as the sibship diagrammed (Supplementary Table S7), have clearly identifiable paternal X- and Y-linked alleles. These markers show the same segregation in 9 progeny in sibship 2, but one recombinant male (male 07) was detected (indicated by red arrows at the top of both parts of the diagram).

The QHPG5 family, however, establishes that the male-determining factor must be distal to the 21.3 Mb marker. This family’s dam and sire were sampled from a natural population in the Quare river, in the Atlantic drainage (Willing *et al.* 2010), and the genetic mapping results are shown in Supplementary Table S7. The QHPG5 parents and the small number of progeny (in total, from two full sibships with different dams; sibship 1 included 13 progeny, and sibship 2 had 10 progeny). The genotypes of LG12 microsatellites yielded one recombinant male (male 7, in sibship 2, labeled QHPG5m07r in Supplementary Table S7). This male inherited his father’s X-linked markers for markers in the centromere-proximal part of the chromosome, but Y-linked alleles for markers from about 21 Mb (Figure 3). To define the crossover breakpoint in male 7, the fish in this sibship (and in the QHPG5 sibship 1) were also genotyped for SNPs throughout LG12, using high-throughput genotyping (see Methods and Supplementary Table S7, which shows results for all markers in both sibships).

Before describing these results in more detail, we first discuss the PAR boundary, which must be distal to the sex-determining locus. This boundary was defined in the QHPG5 family based on the most distal gene showing complete co-segregation with sex among all the progeny other than male 7 in both QHPG5 family sibships; this was an SNP at position 25,998,942 bp in the LG12 assembly, whose total size is estimated to be 26.44 Mb (Künstner *et al.* 2017). In sibship 1, 137 LG12 markers informative in male meiosis show complete sex-linkage, while 10 markers from the terminal part of the assembly of this chromosome are partially sex-linked and can definitively be assigned to the small PAR, consistent with previously published results for this family and for seven other guppy families studied (Bergero *et al.* 2019). A slightly more centromere-proximal boundary, 25,194,513 bp, is found in two other, larger families, the LAH family previously described, from a high-predation site in the Aripo river, with 42 progeny (see Supplementary Table S1 and (Bergero *et al.* 2019), and family ALP2B2, from an Aripo low-predation site, with 136 progeny, see Supplementary Tables S8 and S9).

We next defined the proximal boundary of the crossover event in the QHPG5 family sibship 2, which includes the recombinant male 7. This sibship has the same male parent as sibship 1, but a different dam. Figure 3 shows results for both types of markers in sibship 2, which yielded evidence confirming the crossover event suggested by the microsatellite marker results described above. Despite sibship 2’s small size, the crossover is confirmed by twenty markers (16 SNPs and 4 microsatellites), although the data also reveal likely assembly errors, which we discuss in the next section.

In sibship 2, 141 markers appear to be sex-linked, with all female progeny inheriting the same paternal allele, which is therefore the sire’s X-linked allele, and three males all inheriting the other, Y-linked, paternal allele. However male number 7 is recombinant. He inherited the paternal X-linked allele for the first 111 markers (with 12 exceptions, discussed below), but the paternal Y-linked allele for 21 markers informative in male meiosis; 14 of these markers are in positions from 21,049,596 to 23,293,233, and seven from 24,829,827 to 25,998,942 bp. The 10 terminal markers appear to be partially sex-linked, as in the other families. The male-determining region must therefore be distal to position 21,049,596 in the assembly, and centromere-proximal to the position of the first PAR marker.

The two recombinant individuals suggest that 2/721 = 0.28% of meiotic products in males had crossovers outside the pseudo-autosomal, recombining region. This rate is similar, within the limitations of our modest sibship sizes, to the values obtained previously for recombination between the male-determining factor and the *Sb* coloration factor in a sample of males from the Aripo river; males from a high-predation site yielded a rate of 0.09%, *vs.* 1.3% for low-predation site males (Haskins *et al.* 1961).

### Detection and correction of assembly errors in LG12: the PAR

We next discuss information from which we can infer the correct order of some of the markers, and determine which PAR marker is the most proximal of those so far mapped. Crossing-over occurs at a high rate in the physically small guppy PAR (Bergero *et al.* 2019), allowing us to order the markers by genetic mapping of the region. We also mapped microsatellites within two unplaced scaffolds that are located near one end of the assembly of the homologous chromosome in the closely related fish, *X. maculatus* (see Methods), revealing that they are part of the guppy PAR. The most proximal PAR marker so far mapped is in the unplaced scaffold, NW_007615023.1, while the NW_007615031.1 scaffold maps terminal to the microsatellite markers mapped previously.

**Figure 4:**
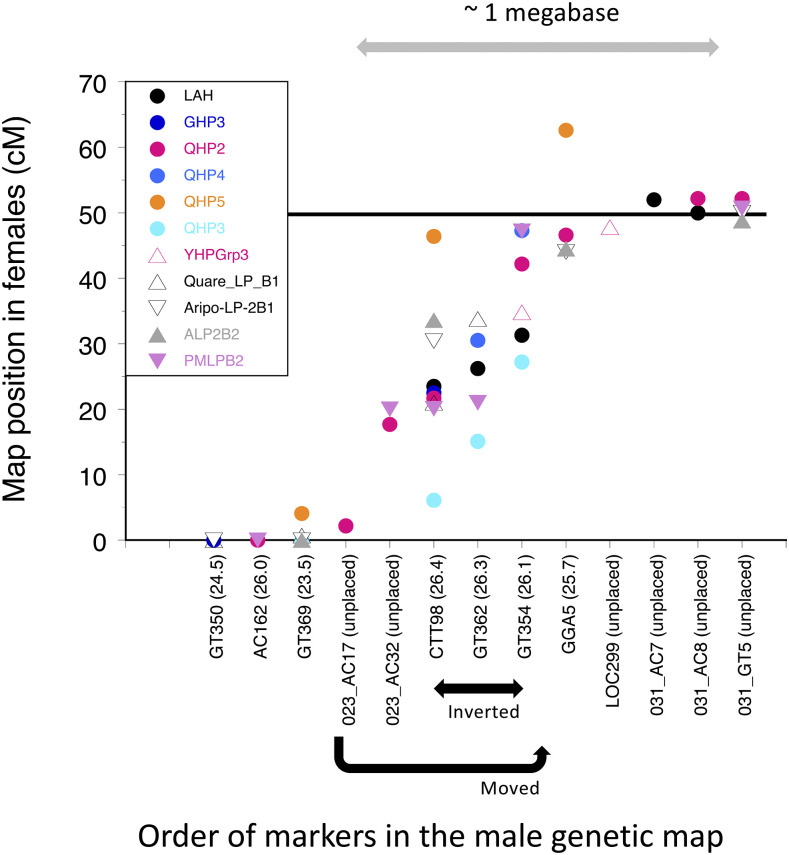
Genetic map of the guppy PAR, based on male meiosis with multiple families with sires from natural populations, in Trinidad (seven from high-predation sites and four from low-predation sites, see Supplementary Table S1). The different color dots indicate different families. Each family’s dam is from the same population as the sire. Only PAR markers are shown, and all more proximal markers co-segregate with the progeny individuals’ sexes (see Supplementary Figure S1), apart from the single recombinant male in the QHPG5 family (see Figure 3 and the text), and a single female in family PMLPB2 (see text). Note that the marker map order in male meiosis is shown, not the positions of markers on the chromosome in megabases (because the errors in the assembly of this region mean that the true positions are not known).

We were able to order the microsatellite PAR markers because some of them were mapped in several different families, and showed consistent ordering that differed from the guppy assembly, suggesting that this region includes several assembly errors (Figure 4). These errors make it impossible to relate the genetic to the physical map, and therefore the figure simply orders the markers according to their genetic map positions. Based on the total size in the published assembly, plus the two unplaced scaffolds, totalling about 815 kb, that we inferred from the *X. maculatus* assembly and mapped to the guppy PAR (see above), our results do not change the conclusion that the guppy PAR cannot be larger than a couple of megabases (Bergero *et al.* 2019). Assuming that consistent high intronic high GC content of a region reflects a high recombination rate that causes GC-biased gene conversion (see Charlesworth *et al.* 2020), the uniformly high GC content (Supplementary Figure S2) of the scaffold that our map reveals to be terminal (Figure 4) suggests that recombination rates are uniformly high throughout the guppy PAR, rather than being clustered into parts of the PAR with very high crossover rates.

### Correction of assembly errors in the non-PAR part of LG12

Genetic mapping cannot order markers in genome regions where crossovers do not occur, or occur very rarely. In guppy male meiosis, markers in the LG12 centromere-proximal region of at least 24 Mb generally co-segregate perfectly with the sex-determining locus (Supplementary Figure S1), making it difficult to map variants within this region. However, if crossovers are infrequent, then errors in the ordering of markers can be detected in rare recombinant individuals, because co-segregation of such markers with others can reveal their true physical locations. Our sibship that includes the recombinant male suggests several discrepancies in the order of sequences within the proximal region of at least 24 Mb of the guppy LG12 that largely co-segregates with the sex-determining locus (see Supplementary Table S7).

The region just proximal to the PAR includes two sets of markers in the recombinant male suggesting assembly errors (Figure 3, Table 2 and Supplementary Table S7). We first discuss nine SNP markers informative in the sire of this sibship labeled Discrepancy 1 in Table 2. These are in LG12 assembly positions between 24,166,053 and 24,508,654 bp (a span of 342,601 bp, including SNPs in four separate genes mapped in this sibship). However, they clearly appear to belong more proximally than the surrounding markers, as their segregation patterns in the recombinant male are identical to those for 90 of the 93 more proximal informative markers. The only exceptions to this pattern are three SNPs at positions between 2,952,176 and 2,953,254 (Discrepancy 2 in Table 2). These three were included in our genotyping experiment because their genotypes in a set of 10 males and 6 females whose complete genomes we sequenced (see Methods) suggested complete sex-linkage (Bergero *et al.* 2019). Their genotypes in the recombinant QHPG5 family male clearly indicate that they are located terminal to the recombination breakpoint that is supported by the 20 markers (16 SNPs and 4 microsatellites mentioned above), where the recombinant male inherited X-linked alleles. They cannot be located more precisely, because markers in this region co-segregate in this small sibship.

**Table 2:** Summary of genotypes in the QHPG5 sibship 2, which includes a recombinant male, for markers that are informative in male meiosis. The two discrepancies are discussed in the text, and the complete genotype information for these markers in both QHPG5 sibships is in Supplementary Table S7. The table shows only markers that co-segregated with the phenotypic sex in all progeny other than male 07, and in all other families where the genotypes were informative in male meiosis. When the recombinant male inherited his sire’s Y-linked allele, his genotype is listed as male in the second column, and it is listed as female when he inherited his sire’s X-linked allele

Description of marker results	Genotype of male 07	Number of markers	Marker positions in guppy assembly (bp)
Centromere-proximal markers	Female	5	458,883 – 2,756,939
SNPs that map more distally (true location more terminal, Discrepancy 2)	Male	3	2,952,176 – 2,953,254
XY markers except in male 07	Female	75	All markers until 20,599,360
XY markers except in male 07	Male	10	21,049,596 – 23,293,233
Markers suggesting mis-assembly (true location more proximal, Discrepancy 1)	Female	9	24,166,053 – 24,508,654
XY markers in all progeny, including male 07	Male	6	24,829,827 to PAR boundary

To further understand the gene order differences between the high-throughput genetic map results for the QHPG5 family and the guppy LG12 assembly, we searched the assembly of the homologous platyfish chromosome (chromosome 8) to find the positions of the four genes with the 9 SNPs that constitute Discrepancy 1 in Table 2 and Figure 3. This reveals that two of these are within a region that is assigned a slightly more proximal location in the platyfish assembly (near 15 Mb). This is near the breakpoint (at 15,847,876 bp) of a small region whose order in the genetic maps of all guppy families with informative markers is inverted relative to the published assembly (see Supplementary Table S10). We conclude that the genetic map arrangement based on the guppy QHPG5 family (with these SNPs more proximal than in the guppy assembly) is probably correct, and that these genes should be re-assigned to a slightly more proximal location. The comparison with the platyfish chromosome 8 assembly also indicates that a set of guppy genes that is assembled in megabase 10-11 correspond to ones that are at the terminus of the platyfish chromosome. The AC162 microsatellite marker was based on sequence in this region, but its genetic map location is around 30 cM in female meiosis in the ALP2B2 family, consistent with a location more distal than 11 Mb; this marker has so far been mapped only in this family.

Our families also support the suggestion in Table 2 that the markers in the LG12 region near 3 Mb labeled Discrepancy 2 are located more distally. The evidence for this conclusion is that the segregation patterns of SNPs that are heterozygous in female meiosis in the dams of three sibships match those of distal markers in the families: SNPs with similar segregation patterns to those of these three SNPs are all distal to position 20,516,823 bp in the LAH family, to 22,002,472 bp in two informative ALP2-B2 family sibships, and to 21,344,716 bp in the QHPG5 family sibship 1. The segregation pattern observed is illustrated in Table 3, together with our interpretation that there is a duplication, which can explain why all males appear to be heterozygous. Furthermore, we examined the segregation patterns of these SNPs in female meiosis in three sibships whose female parents are also heterozygous. The results show that the variants genotyped are located among markers that are close to the PAR boundary, very distant from their assembly positions (Supplementary Tables S8 and S9 show the LAH family and the two informative ALP2B2 sibships, respectively; the same can be seen in the smaller QHPG5 family sibship1 in Supplementary Table S7).

**Table 3:** Example from the LAH family of segregation results found for all three SNPs named “Discrepancy 2” (see text and Table 2). The LAH family sire is inferred to be heterozygous for all three markers. The example shown is for the LG12 site at 2,953,218 bp. The same pattern was observed whenever both the sire and dam had heterozygous genotypes

Dam genotype	Female progeny			Sire genotype	Male progeny		
A/G	A/A	A/G	G/G	A/G	A/A	A/G	G/G
Observed numbers	6	11	0		0	25	0
Interpretation							
X^A^/X^G^	X^A^/X^A^	X^A^/X^G^	X^G^/X^G^	X^A^/Y^G^Y^A^	Not expected	X^A^/Y^G^Y^A^ X^G^/Y^G^Y^A^	Not expected

## Discussion and Conclusions

Dor *et al.* (2019) suggested the “growth arrest and DNA damage inducible gamma-like” (*GADD45G-like*) as a plausible “candidate gene for its role in male fertility”, despite finding “no sex difference in either the genomic sequence or gene expression”. However, several of their observations are difficult to reconcile with this conclusion. First, they state that “the male-specific allele was identified in only 85% of males, but not in females”, and that their family D suggested a possible environmental effect of elevated temperature leading to sex reversal, or recombination that produces inviable recombinant females. Furthermore, the *gu1066* marker identified 97 females as genetically XY, suggesting that sex reversals occur; when mated with normal XY males, these should yield 25% females, but only 19% of the progeny had ovaries, and only four live fingerlings were produced (by one sex reversed female), three XY and one XX. We detected sex-linkage of other SNPs in this gene in the QHPG5 and ALP2B2 families, but this shows only that this gene is on LG12 and not physically distant enough from the sex-determining locus to recombine frequently.

Our analysis of genotypes in natural guppy populations (where recombination in past generations can distinguish between complete and partial sex-linkage) leaves it uncertain whether or not the gene Dor *et al.* (2019) propose as the guppy sex-determining candidate gene is fully sex-linked. A variant in this gene is the first that has been found to be associated with the male phenotype in natural population samples large enough to exclude chance associations (our samples mostly included at least 24 fish, or 48 alleles, per population, see Table 1), and the first association detected in multiple guppy populations, as well as in the strains studied by Dor *et al.* (2019). We detected the same variant in males from six natural populations, derived from both the Caroni and Atlantic drainages (though the variant was not detected in the Northern drainage, Supplementary Table S5). In all six populations where the variant was present, it was found only in male individuals, and was absent in all females (Table 1). All males with the variant were heterozygotes; this is consistent with sex-linkage, but does not provide strong support, because the variant was rare among males in most populations (Table 1), and its overall mean frequency was only 8%. The association is therefore incomplete. We next discuss possible reasons for an incomplete association.

A first possibility is that the variant could have arisen in a fully sex-linked gene (including in the sex-determining gene itself) so recently that it is still segregating in some populations, and has not yet spread to the Northern drainage populations. However, a very recent mutation seems unlikely, given that it is found in two of the three river drainages, which are quite isolated, based on evidence of strong differentiation at microsatellite and other putatively neutral markers (*e.g.*, Suk and Neff 2009; Willing *et al.* 2010; Fraser *et al.* 2015). This variant has therefore probably been maintained for a considerable evolutionary time.

A second possibility is that the variant might be fully sex-linked, but is absent from some males because Y-linked region is organized into several haplotypes, and that the variant is present on one haplotype, but not others. This could also account for the repeated failure to discover fully Y-linked variants in the guppy (see Introduction). None of the variants will then exhibit the genotype configuration expected under sex linkage, but different variable sites will be heterozygous in males with different haplotypes; moreover, each variable site could share the same variant as the X in certain haplotypes, producing male homozygotes, as observed for the variant we genotyped. At the sex-determining gene itself, only Y-and X haplotypes are expected, but multiple haplotypes could be found across a more extensive region, given that Y-linked male coloration polymorphisms are maintained in guppy populations (see Introduction). However, our current data do not support this scenario, as, of 17 other markers in the region from 24.16Mb to 25.32M, including the *gu1066* microsatellite, none have variants consistently associated with maleness.

We also cannot currently exclude a third alternative, that the association reflects a variant in a partially sex-linked gene that recombines rarely with the sex-determining gene. Given the evidence for incomplete associations of variants with the sexes of individuals recently reported for many parts of the guppy sex chromosome pair (Wright *et al.* 2017; Bergero *et al.* 2019; Almeida *et al.* 2020), this alternative remains plausible, especially as the variant shown in Table 1 is assembled close to the genetically determined PAR boundary. Currently, analyses of the guppy sex-determining gene are hampered by the absence of a genome sequence assembled from a male. If the Y-linked region include sequences that are missing from the female genome, these will be mapped to homologous sequences elsewhere in the genome, incorrectly suggesting sex-linkage in other genome regions. Our genetic analysis indeed detected such a situation, which we named Discrepancy 2. However, the guppy Y-linked region need not carry genes that are missing from the X chromosome, as other explanations are possible for the discrepancy we detected between the locations (near 3 Mb) of three sites whose variants had sex-linked genotype configurations in one sample of the two sexes, and the location where they map genetically (near 20 Mb).

Dor *et al.* (2019) concluded that the guppy sex-determining region is a region of 1.26 Mb that is duplicated between LG 9 and 12 and includes 59 LG12 genes, 17 of which had multiple copies; 8 of these had copies on LG 9 as well as 12, while 9 had multiple copies on LG12. The duplicated region occupies 0.43 Mb at about 25 Mb on LG12, and 6 of the 11 genes in the region have multiple copies. We too found a marker that had been assigned to LG9 (the microsatellite GT443) but behaves as fully sex-linked in the male parent of one of our mapping families, LAH (Bergero *et al.* 2019). Its LG9 location is 8.0 Mb, not close to the LG9 region at 17 Mb identified by Dor *et al.* (2019). Thus the LG9 copy could represent mis-annotation rather than the duplication Dor *et al.* (2019) identified. None of our LG9 markers that are informative in male meiosis show sex-linkage in our families (data not shown).

Genes that have moved onto the guppy LG12 since the divergence of the guppy from the platyfish should be found on platyfish chromosomes other than Xm8, the homolog of the guppy LG12. We therefore also examined genes assembled on the platyfish chromosome homologous to the guppy LG9 (see Methods). These genes are mostly carried on Xm12, with a few on chromosome 11 (Supplementary Figure S3A). We inspected the plots for these chromosomes, to determine which guppy chromosomes carry them, and whether some of them are LG12 genes in the guppy. Some platyfish chromosome 11 genes are detected on guppy LG9, but most of them are on LG14 (Supplementary Figure S3C). Platyfish chromosome 12 genes are therefore most relevant for asking whether they form a duplicated region on guppy LG12.

Xm12 genes are (as expected) mostly found on guppy LG9, with a few on unplaced scaffolds, but some are indeed detected on LG12 (Supplementary Figure S2). Six genes, at 6.7, 8.7, 17.0, 18.0, 18.1, and 19.2 Mb of LG12 in the female guppy assembly, are found within a ∼500kb region between 1 to 1.5 Mb of Xm12, intermingled with twenty genes that map to a *P. reticulata* unplaced scaffold, NW_007615028.1 (Supplementary Figure S5). Of the 10 other genes in this unplaced scaffold, 8 have no reciprocal best hits, but two are on Xm8 (as expected for LG12 genes; these are at 6.8 Mb and 19.3 Mb of Xm8). This unplaced scaffold may therefore be part of the guppy chromosome 12. If so, the 26 Xm12 genes may have moved to LG12 from the ancestral LG9/Xm12 chromosome in a single event, followed by rearrangements that led the six genes in the current LG12 assembly to be in two distinct locations (Supplementary Figure S5). There is no compelling correspondence with the region identified by Dor *et al.* (2019). The gene order in the centromere-proximal 20 Mb of the 26.4 Mb guppy LG12 is very similar to that in the homologous platyfish chromosome (Xm8, see Supplementary Figure S4). The large inverted region is an assembly error, as the guppy genetic map supports a gene order in agreement with the platyfish assembly (Supplementary Figure S2, unpublished results of B. Fraser *et al.* 2020, and Supplementary Figure 1 of Darolti *et al.* (2020). Therefore, the location for this set of genes must be distal to the repeat region just after 20 Mb that is prominently visible in Supplementary Figure S4. The figure also shows that the LG12 assembly terminal to this region is either rearranged, relative to the platyfish homolog, or, in parts, remains uncertain.

Overall, however, the results described here suggest that, even if linkage is partial, the region identified by Dor *et al.* (2019) may be very closely linked to the sex-determining locus. Our new results support all previous results, including those from the captive material studied by Dor *et al.* (2019), suggesting that the guppy male-determining factor is located within a region between about 21 and 25 Mb (in the current assembly). We describe the first genetic data based on localizing X-Y crossovers within families. The recombinant male individual indicates that the male-determining factor is indeed located distal to 21 Mb. The recombinant female indicates a location distal to 11.7 Mb, but we lack markers to define the location precisely. Moreover, the 11.7 Mb region in the female assembly is near a breakpoint of the inverted region, whose other breakpoint is near 20 Mb (see above). Testing whether this marker’s actual location is more distal will require an improved assembly. Our results also define the PAR boundary at about 25 Mb (though assembly errors make it difficult to define its boundary or size precisely). Given these problems, and the rarity of recombinant progeny, it remains difficult to define the male-determining locus more accurately. If a more reliable assembly can be generated by long-read sequencing, the rarity of recombination events may still make it impossible to define the locus genetically, though this approach should narrow the region down enough to suggest candidate genes for further testing.
